# ERCP-induced duodenal perforation successfully treated with endoscopic purse-string suture: a case report

**DOI:** 10.18632/oncotarget.4079

**Published:** 2015-06-08

**Authors:** Quanpeng Li, Jie Ji, Fei Wang, Xianxiu Ge, Junjie Nie, Boming Xu, Xiuhua Zhang, Guobing Jiang, Lin Miao

**Affiliations:** ^1^ Medical Center for Digestive Diseases, The Second Affiliated Hospital of Nanjing Medical University, Nanjing, Jiangsu, China; ^2^ Liver Transplantation Center of the First Affiliated Hospital and State Key Laboratory of Reproductive Medicine, Nanjing Medical University, Nanjing, Jiangsu Province, China

**Keywords:** duodenal perforation, endoscopic purse-string suture, endoscopic retrograde cholangiopancreatography

## Abstract

Duodenal perforation is one of the most serious complications of endoscopic retrograde cholangiopancreatography (ERCP) and is difficult to manage. Recently, endoscopic purse-string suture, using endoloops with endoclips, is a relatively new technology and has provided good clinical results. However, the study and use of endoscopic purse-string suture on duodenal perforation is less and its feasibility and safety are unknown. Here, we report a case of ERCP-induced duodenal perforation successfully treated with endoscopic purse-string suture. During ERCP, fluoroscopy revealed abnormal perinephric gas shadowing after breaking and extracting the stones with a stone-removal basket. Then duodenal endoscopy showed an approximately 2.0 cm × 1.5 cm perforation on the lateral duodenal wall, with visible retroperitoneal loose connective tissue. Titanium clips were used to attempt closure of the perforation but failed because of the long diameter of the injury. Therefore, an endoscopic purse-string suture, using endoloops with endoclips, was employed with an Olympus double-lumen endoscope. The perforation was successfully closed. At the 2-month follow-up visit, the patient had no complaints or symptoms. Our case once again proved its feasibility and safety and provided a new perspective for surgeons.

## INTRODUCTION

Endoscopic retrograde cholangiopancreatography (ERCP) is currently the first choice for the clinical diagnosis and treatment of biliary and pancreatic diseases. However, while ERCP has been widely applied in the outpatient setting due to physicians' proficiency in this technique, the incidence of postoperative complications remains high. Duodenal perforation is one of the most serious complications of ERCP, with an incidence of approximately 1% and a mortality rate of 4.2%–29.6% [1]. Therefore, diagnosing the perforation as early as possible, assessing the extent of injury, and selecting appropriate treatment options are significant concerns.

According to Stapfer et al [2], duodenal perforation is classified into the following four types: type I, perforation of the medial and lateral duodenal wall, caused by improper endoscopic technique, which is usually large and can easily lead to intra-abdominal or retroperitoneal leakage so that immediate surgery is necessary; type II, periampullary damage, which should be diagnosed by upper gastrointestinal imaging or CT scan to determine the extent of leakage; type III, involving the distal bile duct, usually caused by the guidewire or stone basket and resulting in a small perforation; and type IV, microperforation with retroperitoneal gas accumulation as the only manifestation, requiring nonsurgical treatment in most cases.

Until now, the optimum treatment methods for duodenal perforation have not been established. Usually, nonsurgical or conservative management is preferred for type II and III injuries. However, this decision should be cautiously made, as nonsurgical treatment failure can cause serious and even life-threatening complications.

## CASE REPORT

**Figure 1:**
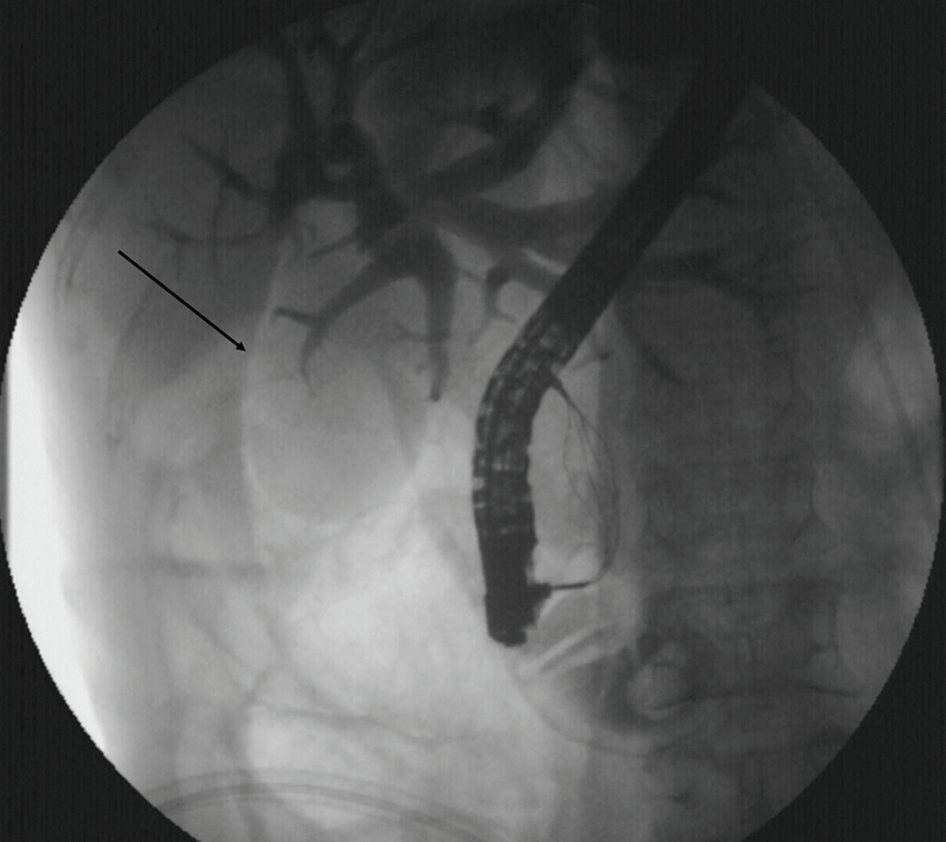
Abnormal perinephric gas shadowing (arrow)

**Figure 2:**
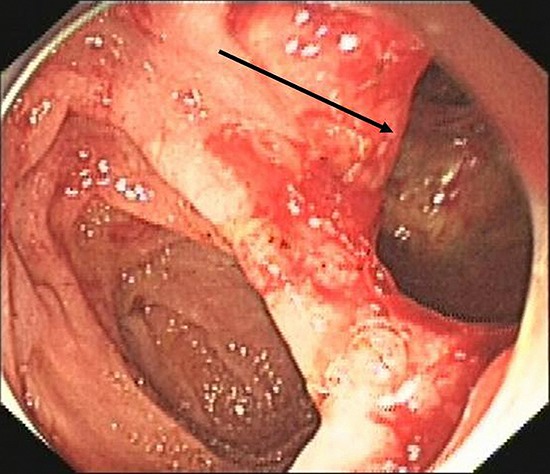
Retroperitoneal loose connective tissue (arrow), viewed endoscopically

A 49-year-old female was admitted for persistent abdominal pain and fever of 1 week's duration. MRCP showed multiple common bile duct stones with accompanying duct dilatation. After admission, antibiotics were administered and ERCP was performed the following day. After successful intubation, ERCP revealed choledochectasia with multiple stones (maximum diameter 1.6 cm × 1.5 cm). After routine EST, papillary sphincter dilatation was performed. Due to the larger size of the stones, lithotripsy and a stone-removal basket (Boston Scientific, Marlborough, MA, USA) were used to break and extract the stones. Repeat cholangiography demonstrated no residual stones; however, abnormal perinephric gas shadowing was observed (Figure 1). Under the duodenal endoscopy, an approximately 2.0 cm × 1.5 cm perforation was visualized on the lateral duodenal wall, with visible retroperitoneal loose connective tissue (Figure 2). We suspected that because the stone basket was over-rigid, it could have penetrated the contralateral intestinal wall due to inertial forces during stone removal.

**Figure 3:**
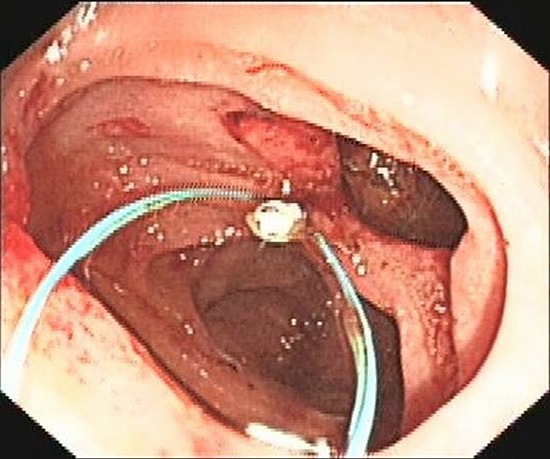
Nylon string and first titanium clip

**Figure 4:**
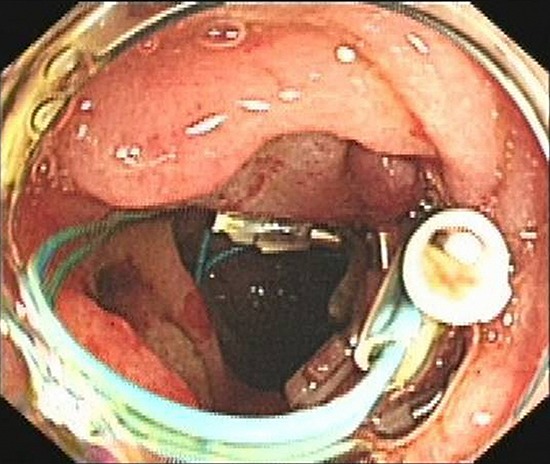
Complete closure of the perforation

An Olympus double-lumen endoscope (Olympus, Center Valley, PA, USA) was used to attempt repair; however, the diameter of the injury was too long. A titanium clip was used to attempt closure of the perforation, but this technique failed. Therefore, an endoscopic purse-string suture was employed. First, the double-lumen endoscope was used to observe the location, size, and appearance of the injury. Then, the following steps were taken: (1) a nylon ring and a titanium clip were inserted along the double channels of the endoscope; (2) after adjusting the angle and location of the nylon ring and titanium clip, the first titanium clip was used to hold the distal end of the nylon ring vertically, firmly approximating the normal mucosa at the distal edge of the defect, and fixing it by deploying the clip (Figure 3); (3) several titanium clips were placed along the nylon string, around the perforation; (4) the last clip was inserted to hold the proximal end of the nylon ring, approximating and fixing it to the normal mucosa at the proximal edge of the defect; and (5) the nylon ring was retracted to draw the distal and proximal edges of the mucosa of the wound together. Endoscopy demonstrated that the perforation had been successfully closed (Figure 4). Postoperatively, a jejunal feeding tube and gastrointestinal decompression tube were placed; she was maintained on bowel rest and placed on antibiotics. Two days later, her abdominal pain was relieved, her abdominal tenderness was significantly reduced, and no rebound tenderness was demonstrated on examination. Subcutaneous emphysema was absorbed after 5 days. Enteral nutrition was initiated 5 days postoperatively. As there was no free intraperitoneal air on plain radiographs 10 days postoperatively, the gastrointestinal decompression tube was removed. After 3 weeks, the jejunal feeding tube was removed and the patient was discharged. At her 2-month follow-up visit, she had no complaints or symptoms.

## DISCUSSION

ERCP-induced perforations primarily occur after EST. Their occurrence may be related to small papillae, a large sphincterotomy, misdirection of the sphincterotomy incision, juxtapapillary diverticulae, or the presence of a Billroth II subtotal gastrectomy [3, 4]. Occasionally, a duodenogastroesophageal perforation occurs during intubation, or cholangiopancreatic leakage after guidewire insertion and biliary tract dilatation, as well as common bile duct perforation caused by lithotomy. In this case, intraperitoneal perforation was caused by an over-rigid stone basket. With regards to ERCP-related perforation, surgery remains the gold standard in the past. While in recent years, endoscopic trials on perforation treatment have been increased. Techniques such as endoclips [5], over-the-scope clips [6], and endoscopic purse-string suture [7] have been reported in case reports. The outcomes supported beneficial role in the closure of duodenal perforation. However, no one particular technique has beend proven efficacy or greater reliability over other closure modalities. Endoscopic purse-string suture, using endoloops with endoclips, is a relatively new technology and has provided good clinical results. However, the study and use of endoscopic purse-string suture is less. We successfully treated an ERCP-induced duodenal perforation with an endoscopic purse-string suture, thus once again proving its feasibility and safety and providing a new perspective for surgeons. Early diagnosis is crucial for the treatment of ERCP-induced duodenal perforation: the perforation must be found during endoscopy and confirmed by postoperative CT scan. Thus, in addition to surgical management, minimally invasive endoscopic techniques including titanium clips and purse-string suturing should be advocated.
